# Trustingly bewildered. How first-year medical students make sense of their learning experience in a traditional, preclinical curriculum

**DOI:** 10.1080/10872981.2018.1500344

**Published:** 2018-08-01

**Authors:** Edvin Schei, Ruth E. Johnsrud, Thomas Mildestvedt, Reidar Pedersen, Stefán Hjörleifsson

**Affiliations:** a Department of Global Public Health and Primary Care, University of Bergen, Bergen, Norway; b Centre for Medical Ethics, Institute of Health and Society, University of Oslo, Oslo, Norway

**Keywords:** Undergraduate medical education, focus groups, relevance of curriculum, quality of teaching, physician role

## Abstract

**Background**: Traditional preclinical curricula based on memorization of scientific facts constitute learning environments which may negatively influence both factual understanding and professional identity development in medical students. Little is known of how students themselves experience and interpret such educational milieus.**Objective:** To investigate first-year medical students’ view of the physician role, and their perception of the relevance and quality of teaching in a science-based preclinical curriculum.

**Design**: Focus group interviews with thematic text analysis.

**Results**: Students portrayed the good physician as communicative, humble, and open, combining biomedical knowledge and moral strength. When asked how medical school supported the development of such characteristics, two partly contradictory discourses emerged. The critical discourse identified decontextualized knowledge, poor pedagogy, lack of critical thinking, and contact with faculty. Students who voiced critical comments also articulated trust that the system would provide the competence they needed, that basic biological knowledge is needed before clinical practice, and that being on your own conveys freedom and responsibility, and helps you grow up.

**Conclusion**: Trust in the educational system, within a substandard learning environment, created cognitive dissonance that students resolved through rationalization, whereby they negated that factual overload and lack of relevance, reflection, and personal feedback was problematic. The cost of this mechanism is possibly that inferior teaching is perceived as normal, necessary, and good enough. If so, these future physicians’ ability to critically evaluate and create quality in medical education and practice, may be weakened.

## Introduction

In this paper, we present an interview study of medical students’ perceptions of their learning environment and educational progress, at the end of their first year in a traditional medical school, where education largely consists of basic science didactic teaching, and written exams. The aim of the study was to understand how first-year medical students experience and interpret the educational experience, and its effects on them, in light of their ideals of the good physician.

Learning to become a physician is a process of change, where not only the student’s knowledge and understanding of medical problems evolve, but the student as a person is altered in the process of developing a professional identity [1–4]. Medical socialization affects judgment, morality, self-perception, emotional, and social functioning [5]. The change occurs over years of participation in a community where trainees interact with peers, teachers, patients, and medical role models. The medical school experience is constituted by cultural practices encompassing both the explicit, the informal, and the hidden curricula of medical training [6–9]. Participation in this ‘community of practice’ [10] fosters a dynamic student identity that evolves over the years. This identity is influenced by the cognitive input of explicit teaching practices, but even more by each individual’s largely unconscious internalizations of the behaviors, language, and conceptions of knowledge and quality inherent in medical teaching, clinical practice, and the informal aspects of academic and student environments [3,8,11–13]. The evolving medical student identity is the stem from which develops the future physicians’ full-fledged professional identity, capability and outlook, embodying the tacit norms of physicianship [14,15], of good and right medical actions [16,17], of what it means to be ‘a real doctor’ [18,19].

A number of studies have reported negative development of core professional attributes such as empathy and patient-centeredness during medical school, including in the first year [4,6,20–24]. It is also well documented that medical students have a higher prevalence of burnout, depression, and suicidal ideation than comparable adult populations [25–28]. The first year of a traditional medical school is a challenging passage that entails both explicit and tacit emotional and cognitive adaptations, many of which are unintended, and potentially unfavorable for the future physicians’ ability to carry out ‘good and right healing actions’ [17,29].

### Study aim

Based on the above literature, we theorized that the discourse of contemporary medical students at the end of year 1, i.e., their thoughts, judgments, stories, decisions, and ideals of medical education and practice, is strongly influenced by the medical school experience and the learning environment they find themselves in. Knowing more about this discourse may increase our understanding of the mechanisms and formative power of the hidden curriculum, and possibly help prevent burnout and depressive symptoms in medical students. It may also contribute to the design of curricular and teaching improvements. We therefore decided to do focus group interviews with a sample of medical students at the end of their first year in a Norwegian medical school with a traditional 2-year preclinical period followed by 4 years of clinical training. After the study was carried out, a new curriculum has been implemented. Follow-up studies are planned.

We wanted to understand how beginner students describe, appraise, and judge the relevance of the learning and development they undergo in a traditional medical school, where many of the above-mentioned formative processes are likely to occur. What do present-day students think about the competence required of the ideal physician, and how their education helps them learn and develop what they will need? Do they identify gaps between ideals and reality? Do they share similar views, or are their experiences and interpretations individually divergent?

## Material and methods

### Participants and data collection

Participants were recruited among 160 medical students towards the end of year 1. Two focus groups of eight participants were deemed to provide sufficient information power for an exploratory analysis, where the ambition was not to cover the entire array of phenomena, but to discern patterns relevant for the study aim [30]. After initial analysis, the two groups were found to provide similar information, hence no further data collection was made.

A large number of students volunteered to participate following a brief introduction by ES, after a lecture in anatomy. The first sixteen in line, coincidentally eight males and eight females from a class consisting of approximately 70% females, were organized in two groups of eight, with equal gender distribution. Interviews were carried out the subsequent week, each lasting approximately 1.5 h. The interviews were audio-recorded and transcribed verbatim. The moderator (RP) used a semi-structured interview guide to explore students’ thoughts about the ideal characteristics of physicians, how the university prepares them to be good doctors, and their thoughts about qualities and deficiencies of the first year of medical school.

The first-year curriculum consisted of 858 h of planned teaching, of which 73% covered basic biological sciences and anatomy. The remaining sessions covered statistics, ethics, Latin, introduction to philosophy, and a 3-h introduction to humanistic medicine. There were no teaching elements dedicated to introductory orientation, patient contact, hospital visits, mentoring, or individual supervision and feedback, and very limited expectations of structured critical reflection.

The authors are physicians, RP working in medical ethics research, the others as family physicians and educators. ES is responsible for ‘medical professionalism’ in the reformed curriculum that was introduced after the current study was carried out.

### Data analysis

The transcripts underwent a thematic analysis using Malterud’s method of systematic text condensation through the following steps [31]. (1) The material was read several times by the authors, with preconceptions bracketed, to obtain a common overall impression of the topics that appeared to be of interest to the students. (2) Units of meaning representing different aspects of the participants’ experiences and reflections were identified and coded, in an iterative process where categories were changed, merged, deleted, or renamed as new meaning emerged. (3) Within each of the coded groups, meaning was condensed and abstracted, and quotations that illustrate the categories particularly clearly were selected. (4) In the last step, the contents of each coded group were summarized to generate descriptions and concepts reflecting participants’ most prominent experiences and reflections.

### Ethics

The study was approved by the Norwegian Centre for Research Data, the data protection official for research for Norwegian universities.

## Results

The findings are presented without comments, followed by a theoretical discussion. Quotes are followed by a tag indicating group, participant number, and gender.

The students largely agreed on the characteristics of good physicians, underscored the need for solid knowledge, but talked considerably more about the humanistic aspects, such as communication skills, meeting patients with interest and warmth, seeing the physical and the mental as a whole, being humble, admitting error, and generally being a person who has the moral strength to do more than what is expected of the average person.

It’s a profession that expects physicians to make a special effort, do things that are not expected of ordinary people, in a way. That they are strong persons who can tolerate doing things that are morally difficult to do. (A2m)

### Ambivalence and two discourses

When asked whether and how the medical school helped them develop what they needed to fulfill these ideals of their future professional role, two different discourses emerged, one critical and one apologetic. There were no signs of tension or established lines of discord between students who were more or less critical or accepting of the order of things. Several students voiced both perspectives, sometimes within one statement, such as this:

I clearly see the preclinic as useful, but it’s a bit like holding your breath and waiting for the goal. (B7f)

Students mostly agreed on the descriptions, examples, and reflections given by peers, whether the implications were critical or apologetic, but often a critical comment was balanced by an apologetic one, and vice versa. Thus, the conversation weaved back and forth between the two perspectives on the experience and learning outcome of their first year of medical education. It was apparent that many of the topics discussed in the focus groups had not been reflected upon previously. In the following, we have separated the critical from the apologetic discourse, to clarify the contradictory and complementary ideas that constitute the students’ thinking.

#### The critical discourse

The critical discourse exhibits four lines of reasoning: disconnected knowledge, poor pedagogy, lack of reflection, and isolation and intimidation from lack of contact with faculty and staff.

##### 1. Disconnected knowledge

The students found it hard to learn a huge amount of theoretical facts without seeing the ‘links and connections’ to clinical medicine. Even within the basic sciences the links were not made clear: *I mean seeing the connections between chemistry and biochemistry and cell biology, they haven’t even done that (A5f)*. Students had not been told what was most important, and felt they had to figure it out on their own. Not knowing the relevance of the knowledge forced them to focus on the exams. *We automatically focus on what we need for the exams when we have no clue what we’ll need in the clinic (A5f)*. Clinical examples, like knowing how a medicine works, were hugely motivating. Though they saw the need for lots of theory, they missed patient contact, and pointed out that it takes time to change as a person, and learn how to relate to and care for sick people. *I feel that to learn how to be humane is almost as important as learning about disease (A6f)*. What little they had learned about actually working as a physician had been gleaned from student organizations and peers, or from physicians in the family, not the medical school. *If we only could have been with someone and just seen, just heard… Even if it wasn’t a separate subject, just followed someone and just heard a little bit… (B6f)* They felt unable to envision their future functions, how it looks and feels. *I’ve felt like I’ve lied every time I’ve said that I study medicine, that it’s something I kind of make up (B7f).*

The teaching experience was generally devoid of contact with patients and health care professionals, except the teachers who were often full time academics with no or limited clinical experience:

There is a lot of human contact in being a physician, and it surprises me that there’s so much theory and so little human contact. It’s as if they leave that to the students. It’s as if we are being educated to see the human being as a machine, a biological machine. (B2m)

##### 2. Poor pedagogy

Students agreed that many lecturers came ill prepared and unmotivated for teaching, dishing out information on powerpoint slides at high speed without contextualizing the facts or activating the students. *You have this enormous amount of knowledge. Having to filter out something, you can’t absorb it all, or maybe you can, but who does that, in a way (A7m)*. The basic science teaching could be overwhelming and boring.

Basic science is very heavy and theoretical, if you’re not interested in biochemistry and superbasic stuff, it’s a very boring subject (A8f), and the most pacifying teaching I’ve ever experienced is here at the university (B2m).

Many students suspected that they would forget most of the knowledge, or be unable to apply it because of lack of training, passive learning, and lack of understandable relevance and practical application of knowledge.

You see the patterns and you manage to think it when you have to, but you never get any practice. You read for an exam and then the next and then the next and the next. And later you’re not able to get it out. (B2m)

##### 3. Lack of critical thinking

*They teach you to follow a template, there’s little critical thinking and little independent thinking, the way I see it (A2m)*. Students pointed out that medicine is a subject where there can be many answers to a question, and you have to learn to think about the possibilities. Basic science teaching was perceived as serving them a conception of science where answers are right or wrong, with little stimulation for reflection or independent thinking. *Based on what we’ve had so far, there’s one right answer and you’re supposed to get it. That’s the goal, you’re supposed to be good enough to always find the right answer (B6f)*. The quest for right answers, rote learning and compliant thinking had changed their views on physicians’ competence:

I’ve always thought that physicians have solid knowledge, but now I’ve changed my mind in a way. I mean that we are too compliant, that we learn enormously much, but learn very little about thinking by ourselves. And the subject is so important that if they teach you something it’s hard to go against it. (A7m)

Ethics teaching was the exception: *In ethics, the difference was that you were activated and made to think yourself, and then you remember more (B2m).*

##### 4. Isolation and intimidation

Students described themselves as experiencing no personal contact with faculty members, feeling invisible and anonymous. They shared a common fate and daily life as medical students, struggling with the same exams, the unclear relevance of the hard work, their own identity issues, competition, peer perfectionism, and in some cases social isolation. *Relating to others and understanding others is sort of extra hard when you’re trying to figure out who you are, yourself (B4m).*

Entering medical school was described as intimidating, with faculty perceived as distant ‘oracles.’

Once a lecturer said ‘hello’ to me as he entered the auditorium, even if we had never met before. And it gave me a sense of security, like I dare more to reflect on my own, because I see they’re just human. (A3f)

Students saw potential connections between the hierarchy and perfectionism experienced in medical school, and the stereotypical ideal of physicians as somehow superior. *Here it’s very important to be perfect. It’s hard to admit errors in medical school, it’s like if you’re going to be a doctor, an authority figure, you’re supposed to be in control of most things (B6f).*

Many of the students described a feeling of losing their self and personal commitment: *I’ve never felt so anonymous in a school. In the army you’re supposed to be anonymous, but I felt much more uplifted. It’s easy be become part of the mass here (B2m)*. Another aspect of this alienating experience was the loss of individuality and independent thinking:

When you enter medical school it’s maybe a little frightening, and… You’ll accept anything, you don’t dare to object against them (A3f). It’s like you go through a factory, and you end up a product of that factory. (A3f)

Some of the students emphasized that the lack of human contact between teachers and students may be most problematic for the students that do not fit in: *Maybe there’s no need for sort of human contact between teachers and students. But you see students who just seem to be muddling along all alone. Maybe if they had someone to talk to…? (B2m)*

#### The apologetic discourse

Though all students acknowledged that there were problems, most also gave examples and arguments that allowed the first year of medical school to be seen as just what they needed to become good physicians. The arguments fell into three categories: trust in the wisdom of the system, the protective function of knowledge, and the need to grow up and take responsibility.

##### 1. Trust the wisdom of the system

Students voiced many arguments and interpretations in support of the usefulness and adequacy of the education they received.

The profession is so big, and you choose specialty so late, that lots of information has to come in a relatively short time. And that information has sort of a given answer (A7f). That’s in the nature of the subject. (A2m)

They also reasoned that the enormous amount of knowledge one has to learn makes it logical that you just have to start somewhere, even if you don’t see the large picture. *I see the point of patient contact… But I think it’s important to think about basic sciences the first year. I mean, it’s quite a shock when you begin to realize this is such an enormous subject (A4m).*

Poor pedagogy was acknowledged, but not seen as representative of the system. *Not everything needs to be clinically relevant. We don’t’ have to be doctors yet (A2m).*

The informants trusted the basic fairness of the system, that hard work would pay off, and that the relevance of subjects taught would become apparent with time. *They tell us all the time that it’s important later. You learn it later. You find out later what is important (A1m).*

I have this feeling that as long as I do my best… And if that’s not good enough, I won’t become a doctor, but if it’s good enough, then I guess I can be one (A8f).

##### 2. Knowledge protects you

It was often pointed out that basic science knowledge would make it less frightening to talk to patients, and secure one’s ability to function well in the clinical phase of the study. *I think it’s fine to know some anatomy before you sort of have to talk to patients (A5f).*

Of course it must be mostly theory, you’re supposed to be confident in your reasoning and then it’s of course a lot of reading and stuff (B3m).

##### 3. It’s your own responsibility

Many students saw the lack of supervision and interaction with staff as a manifestation of freedom and responsibility. *It’s not the lecturers’ job to say hi to everyone (A7m)*. Students should teach themselves how to be critical to the knowledge transmitted, and to see what the more or less important parts were. *Then I think one sort of has to take the initiative and filter out what one thinks is less important and put in what is more important. You’ve got to take some responsibility yourself (A7m).*

Handling student life without being seen by teachers was a way of growing up. *You’re supposed to be able to get through these studies without being seen, because you have to grow up at some stage (B5f).*

## Discussion

Students readily agreed on the characteristics of a good physician, underscoring the humanistic ideals while also pointing out the importance of knowledge. When asked how the university supported their development towards these competences, two partly contradictory discourses, one critical and the other apologetic, emerged. The critical discourse focused on a lack of clinical contextualization, poor pedagogy, lack of critical thinking, and isolation and intimidation. The apologetic discourse highlighted trust that the system would provide the competence they needed, that basic knowledge is needed before clinical practice, and that lack of close supervision confers freedom and responsibility.

The discourses of criticism and apology seem to reveal a balancing act where the medical students, who may see themselves as fortunate champions of the prestigious and highly competitive race to enter medical school, strive to maintain a positive self-image by constructing interpretations and adaptive strategies that ameliorate the experience of partly disappointing learning trajectories, whose usefulness in relation to their professional goals and ideals is dubious. How these adaptive strategies can be understood, and how they may influence students’ subsequent learning and professional identity development, is discussed using theories of cognitive dissonance and socialization.

### Impact of the learning environment

Based on the syllabus described in the methods section, it seems unequivocal that our informants’ learning environment falls within a tradition of twentieth century medical education that has come under heavy criticism [1,32]. Curricula, largely structured around acquisition of measurable knowledge and skills, disregard insights from education research showing that adult learners need to be engaged in supervised practice, feedback and guided reflection, for factual knowledge to evolve into practice competence [32–39]. Recent publications have concluded that many of the world’s more than 2400 medical schools have ‘fragmented, outdated, and static curricula that produce ill-equipped graduates,’ with mismatch of competencies to patient and population needs, poor teamwork and ‘narrow technical focus without broader contextual understanding’ [32,39]. In their call for reform of medical education in 2010, the Carnegie Foundation for the Advancement of Teaching stated that ‘factual overload’ of students in many medical schools invites ‘learning strategies such as rote memorization that are inimical to scientific reasoning and inquiry’ [1]. This echoes criticisms offered decades ago, that rather than a ‘way of knowing,’ science becomes a world of established ‘facts’ and soluble ‘puzzles’ [40], and that ‘the type of basic science education traditionally provided in medical schools is singularly effective in annihilating the motivation and the idealism of a substantial majority of the students, while still leaving most of them with a quite inadequate scientific base for their later clinical education and for their subsequent practice as a clinician’ [41]. On the other hand, research suggests that experiential learning, such as early patient experiences, by providing relevance and motivation, helps students understand basic science better and develop clinical skills quicker [42].

A learning environment lacking personal supervision and guidance is a risk factor for mental health problems, such as burnout and depression [25,43,44]. A recent meta-analysis indicates that more than 50% of medical students have burnout symptoms [28]. Teaching and assessments perceived as lacking in relevance and meaning, of the kind indicated by our results, increase the likelihood of burnout [25]. Motivated and intelligent persons who expect to derive a certain fulfillment from work are those who are more likely to become disappointed, helpless, and hopeless, and eventually burn out [45]. Burnout, with its typical alienation and cynicism, may be a link to understanding the stunting of empathy and moral reasoning that occurs in many medical students, often starting in the first year [46,47]. We have no data on the mental health of our informants. The apologetic discourse suggests that their morale was upheld by compensatory mechanisms.

Medical students are perfect objects for socialization, writes Hafferty, because of pressure and drive to ‘survive’ and ‘join the club’ [5, p. 59] and to accomplish tasks and goals defined by others. Secondary socialization is the formational processes that transform lay youths to professionals, through tacit and largely unconscious mechanisms of adaptation whereby newcomers relinquish aspects of their former selves and adopt norms that dominate their new environment [5]. Hafferty has previously suggested that socialization can involve ‘internalising norms and values about *not* reflecting on medical work, about *not* thinking too much about certain medical practices’ [48, p. 31] (*original emphasis*).

### The fox and the grapes – dissonance and rationalization

Driven by hunger, a fox tried to reach some grapes hanging high on the vine but was unable to, although he leaped with all his strength. As he went away, the fox remarked ‘Oh, you aren’t even ripe yet! I don’t need any sour grapes.’ [49]

This famous Aesop’s fable illustrates the core of rationalization: rather than admit his failure, the fox convinces himself that the grapes are not really desirable. By denying that he has a problem, he avoids the painful emotions and threatened self-image associated with helplessness, bad luck and unsatisfied appetite. The informants in our study also find themselves in a dissonant position, and solve it by rationalization: they reinterpret the ‘sour grapes’ of the low-quality learning environment they concisely describe, as valuable knowledge and responsibility, an opportunity to grow up, and thus quite sweet, after all. Residual frustrations are balanced by trust in the wisdom of the system: everything will become meaningful at a later stage. In this lies a tacit devaluation of themselves as too inexperienced or ignorant of clinical practice to exert judgment about the relevance of the education they receive.

Several factors may reinforce medical students’ tendency to rationalize. Medical studies are expected to demand sacrifice, and it has been shown that students admitted to medical school undergo anticipatory socialization, adjusting their values and dispositions in the time between admission and entry [50]. Students arriving at university fresh from secondary education are used to learning by memorization [51, p. 213], and see rote learning as natural. Goldie suggests that late modern societal trends may ‘affect students so they become passive acceptors of whatever they find in their day-to-day worlds’ [8]. Moreover, the prestigious reputation of medical studies with its connotations of science, academia, and altruism conveys authority and high moral and intellectual standards, making it even more unlikely that the medical novice should perceive medical education as deficient, or herself as apt to evaluate it ([4, p. 307], [5]). The result is *cognitive dissonance*, the discomfort experienced when a person holds contradictory ideas or beliefs [52]. In this case, the students reduce dissonance by negating that the disconnectedness, irrelevance and factual overload they experience is real, or a real problem. The cost of this mechanism is possibly that these students, like generations of physicians before them, come to perceive inadequate teaching methods as ‘just the right thing,’ which might explain why deep changes in medical education are slow to happen [38].

### Critical remarks

The results are based on interviews with a small number of students, and have limited generalizability. In constructing the interview guide, monitoring the interviews, and selecting and interpreting quotes, the researchers’ conceptions of what constitutes good medical education may have colored the process, potentially introducing a bias.

## Conclusion

What becomes visible through the discourse of our informants are unintended learning processes whereby students alter their views of science, learning, and medicine as such, by partaking in and approving of routine life in medical school, where well founded critical impulses are dampened by rationalization. The result are professional identities and perspectives awash in unacknowledged, and not entirely benign [53], perceptions of knowledge, quality, value and competence. The pedagogy and learning environment described by the informants may be suspected of contributing to impoverished critical thinking, naïve objectivism and deficient understanding both of biomedical science and of how clinicians use knowledge, as well as alienation and suppression of emotion, producing burnout and cynicism. The most precarious aspect of our findings may not be that the students’ cognitions are molded by their experience, but that they are unaware of it and hence prevented from resisting or mitigating it. What we have seen in this study, is a glimpse of educational mechanisms that may weaken future physician’s ability to perceive, evaluate, and reflect critically on quality, in education and medicine.
